# Exploring an activity-enhanced scaffolding for knowledge structure representing: could the knowledge-activity connection promote students’ online learning experience in AI curriculum?

**DOI:** 10.3389/fpsyg.2025.1725612

**Published:** 2026-01-08

**Authors:** Haipeng Wan, Rongping Que, Lingna Cheng, Shan Li, Qi Wang, Jing Liu

**Affiliations:** 1 College of Education, Capital Normal University, Beijing, China; 2 Daxing Branch of Cuiwei Primary School, Beijing, China; 3 Department of Education and Human Services, Lehigh University, Bethlehem, PA, United States; 4 Artificial Intelligence and Human Languages Lab, Beijing Foreign Studies University, Beijing, China

**Keywords:** adaptive learning, knowledge network, knowledge-activity scaffolding, learning outcome, online learning

## Abstract

**Introduction:**

The effectiveness of online learning is often hindered by fragmented content organization and limited guidance, leading to reduced student engagement and learning outcomes.

**Methods:**

This study introduces an activity-enhanced scaffolding for knowledge structure representing that combines dynamic knowledge network visualization with adaptive activity pathways to address these challenges. The approach provides students with explicit connections between learning resources and activities while visualizing course knowledge structures and learning progression. We conducted a four-week quasi-experimental study with 60 middle school students to examine the approach’s effectiveness.

**Results:**

Students were divided into experimental (n = 30) and control (n = 30) groups, with the experimental group using the scaffolded learning environment while studying an artificial intelligence curriculum. Using both quantitative and qualitative methods, we analyzed learning performance through knowledge tests and concept mapping, assessed learning attitudes via questionnaires, and evaluated thinking levels through the Structure of the Observed Learning Outcome (SOLO) taxonomy.

**Discussion:**

Results revealed that the experimental group showed significantly higher scores in knowledge acquisition and knowledge organization. Moreover, experimental group demonstrated more positive learning attitudes across behavioral, cognitive, and emotional dimensions, and achieved higher levels of thinking, with 73.33% reaching relational or extended abstract levels compared to 21.11% in the control group. These findings suggest that integrating knowledge visualization with structured learning pathways effectively enhances online learning outcomes. The study contributes to both theoretical understanding of scaffolding design and practical guidelines for developing more effective online learning environments.

## 1 Introduction

Online learning has become an essential educational approach, offering students flexibility, self-paced learning opportunities, and abundant resources (Fang et al., 2023; Wei and Chou, 2020). While these characteristics make online learning appealing, they also present significant challenges. The absence of structured organization in course content and limited opportunities for meaningful social interaction often led to reduced learning effectiveness (Ateş and Köroğlu, 2024; Dixson, 2015). Additionally, learners frequently experience cognitive overload due to the demands of processing and organizing information independently (Conrad et al., 2022; Jebur et al., 2022).

While various scaffolding approaches exist to support online learning (Song and Kim, 2021; Lange et al., 2023), most focus on providing content-specific guidance rather than addressing the structural organization of knowledge and learning pathways. Traditional scaffolds often fall short in two critical aspects: helping students navigate complex knowledge relationships and supporting systematic progression through learning activities. Students with limited prior knowledge particularly struggle to utilize existing online scaffolds effectively, leading to confusion and potential course abandonment (Kellen and Antonenko, 2017; Jebur et al., 2022).

To address these limitations, we propose an activity-enhanced scaffolding for knowledge structure representing that combines two innovative components: (1) a dynamic knowledge network visualization that reveals relationships between concepts, learning resources, and learner interactions, and (2) an adaptive activity pathway system that provides explicit connections between learning content and activities. This dual-component approach differs from traditional scaffolding by focusing on the organizational structure of knowledge and learning processes rather than just content support. The approach aims to reduce cognitive load (Munshi et al., 2023) while facilitating deeper engagement with course materials.

This study examines the effectiveness of activity-enhanced scaffolding for knowledge structure representing through three research questions: (1) What is the effect of the integrated scaffolding on students’ learning performance? (2) How does the approach influence students’ learning attitudes? and (3) What impact does the approach have on students’ thinking levels? The findings from this study contribute to both theoretical understanding and practical applications in online learning. From a theoretical perspective, our work extends existing scaffolding theories by demonstrating how the scaffolding support can enhance learning outcomes. From a practical standpoint, the results provide guidelines for the design of more effective online learning environments that better support student progression and deeper learning.

The remainder of this article first reviews relevant literature on scaffolding approaches in online learning. We then detail our research methodology, including the design of the integrated scaffolding approach, experimental procedures, and assessment instruments. This is followed by the presentation and discussion of the results. Finally, we offer conclusions and directions for future research.

## 2 Literature review

### 2.1 Theoretical foundation of scaffolding

The concept of scaffolding emerged from Wood et al.’s (1976) research on tutoring processes that enable novice learners to solve problems beyond their independent capabilities. Through appropriate guidance and support, learners can accomplish tasks that would otherwise be beyond their unassisted efforts. This concept fundamentally aligns with Vygotsky’s (1978) Zone of Proximal Development (ZPD) theory, which describes the distance between a learner’s actual developmental level and their potential level achievable through guidance or peer collaboration (Xi and Lantolf, 2021). The ZPD framework emphasizes that a well-designed scaffold must precisely bridge the gap between novice learners’ current abilities and their potential competence (Fang et al., 2021). This theoretical foundation underscores the importance of calibrating educational support to match learners’ developmental needs.

### 2.2 Scaffolding approaches in online learning

Recent years have seen significant advances in educational scaffolding design, with researchers exploring various approaches to support different aspects of the learning process (Lu and Xie, 2023). Multiple scholars have proposed comprehensive scaffolding principles that guide the development of effective support structures (Belland and Kim, 2013; De Backer et al., 2016; Bacca-Acosta et al., 2022; Wang et al., 2021). These principles have been implemented across diverse learning contexts, including problem-solving environments (Liang and She, 2023), inquiry-based learning scenarios (Lämsä et al., 2020; Van Hoe et al., 2024), and online discussion forums (Song and Grace, 2018; Barzilai et al., 2020).

Research has consistently demonstrated the positive effects of scaffolding on student engagement and learning outcomes. Song and Grace (2018) found that diverse scaffolds in online courses significantly enhanced student engagement levels. Different types of scaffolds serve distinct purposes in online learning environments. Procedural scaffolds have proven particularly effective in helping students generate problems and develop solution strategies (Yu et al., 2013). Conceptual scaffolds play a crucial role in supporting emotional regulation and goal mastery, helping students maintain focus and motivation throughout the learning process (Belland and Kim, 2013; Wang et al., 2022). Interactive procedural scaffolds have shown significant benefits for enhancing self-regulated learning, though their impact on metacognitive awareness remains limited (Kellen and Antonenko, 2017).

### 2.3 Knowledge organization and visualization in scaffolding

The effective organization and visualization of knowledge have emerged as a crucial aspect of online learning scaffolds. Communication scaffolds serve as metacognitive tools that help novice learners structure and process information. For example, the Knowledge Forum™ platform (Scardamalia and Bereiter, 1994) implements structured communication scaffolds through message descriptions (e.g., “my theory,” “I need to understand”) to facilitate knowledge construction and collaboration in online environments (Lin and Hou, 2024). Such platforms demonstrate how structured support can guide students in articulating and organizing their understanding.

Moreover, recent advances in knowledge visualization techniques have offered new possibilities for scaffolding online learning. Ma et al. (2021) investigated knowledge graph-based structured scaffolds and found that these tools not only improved student performance but also led to more coherent knowledge structures. Students using these scaffolds demonstrated a clearer understanding of relationships between concepts and could better integrate new information with existing knowledge. Similar findings were reported by Hsu and Hwang (2017), who showed that visual representation of knowledge relationships enhanced both comprehension and knowledge retention.

However, most current visualization approaches face significant limitations. First, they typically remain static and disconnected from learning activities. Wang et al. (2019) found that while visualization of learning behaviors improved performance, static visualizations could not adapt to students’ evolving learning needs. Second, existing tools often focus narrowly on content relationships without considering how students engage with this content through learning activities. Gao et al. (2005) emphasized that effective scaffolds should support both knowledge organization and the metacognitive processes involved in knowledge construction.

### 2.4 Current gaps

The limitations identified in current scaffolding approaches reveal three critical research gaps that need to be addressed. First, existing scaffolds lack dynamic knowledge organization support. While studies have demonstrated the value of knowledge visualization (Ma et al., 2021; Hsu and Hwang, 2017), current approaches typically present static representations rather than interactive knowledge structures that evolve with student learning. Moreover, there is insufficient integration between content and learning activities in current scaffolding designs. Traditional scaffolds often treat content support and activity guidance as separate elements, creating fragmented learning experiences. Although researchers have explored various types of scaffolds for specific learning tasks (Yu et al., 2013; Kellen and Antonenko, 2017), few studies have examined how to create coherent pathways that connect learning content with associated activities. Finally, existing scaffolding approaches provide limited support for developing higher-order thinking skills. Most research emphasizes basic cognitive development and learning motivation (Bacca-Acosta et al., 2022; Parker et al., 2021; Villalonga-Gomez et al., 2023), but fails to address how scaffolding can support the progression from surface-level to deeper understanding.

The complexity of these challenges suggests the need for an integrated approach to scaffolding. While individual studies have addressed specific aspects of online learning support, questions remain about how to design scaffolds that simultaneously address knowledge organization, learning pathways, and thinking development. Our study aims to fill these gaps by proposing and evaluating a comprehensive scaffolding framework that integrates dynamic knowledge visualization with structured learning progression support.

## 3 Design of the activity-enhanced scaffolding for knowledge structure representing system

Our approach addresses the identified research gaps through two interconnected components: (1) a dynamic knowledge network visualization component and (2) an adaptive activity pathway generating component. These components work together to become a whole adaptive learning system and provide comprehensive support for online learning. The system was designed based on the Learning Cell Knowledge Community (LCKC) platform,^1^ building upon the foundational concept of learning cells (Yu et al., 2017). The LCKC contains resource creation module, user interactive module, recommendation module and dashboard module. The knowledge structure representing system of activity-enhanced scaffolding was nested in both dashboard module and recommendation module. The whole workflow is as follows: First, the course teacher utilizes resource creation module to develop the learning content, learning activities, and evaluation scheme. Then, the learner engages with these resources and activities through the user interaction module. Meanwhile, all the processing and resulting data, generated by both the teacher and the learner, are stored in a database to be used for computing the learner’s cognitive state and knowledge structure. Whereafter, the recommendation module calculates similarities by integrating the learner’s cognitive state and knowledge structure, subsequently generating an adaptive activity pathway for each learner. Finally, the dashboard module displays the cognitive state, knowledge structure, and recommended learning resources in a graphical format, providing essential learning navigation and support to the learner. For a more detailed introduction, please refer to the previous research (Wan and Yu, 2020).

In the system, the prior component acts as the evaluator of learners’ learning state. The second component acts as the tutor which could provide learners with suggestions. We designed the components according to learners’ requirements and there were two principles:

1. Learners must first accurately assess their current learning status before implementing targeted strategies to enhance learning performance.
2. Systematic instructional scaffolding becomes essential when learners demonstrate fragmented understanding of disciplinary knowledge structures.

With these principles, the following components were designed.

### 3.1 Dynamic knowledge network visualization component

**Figure 1:**
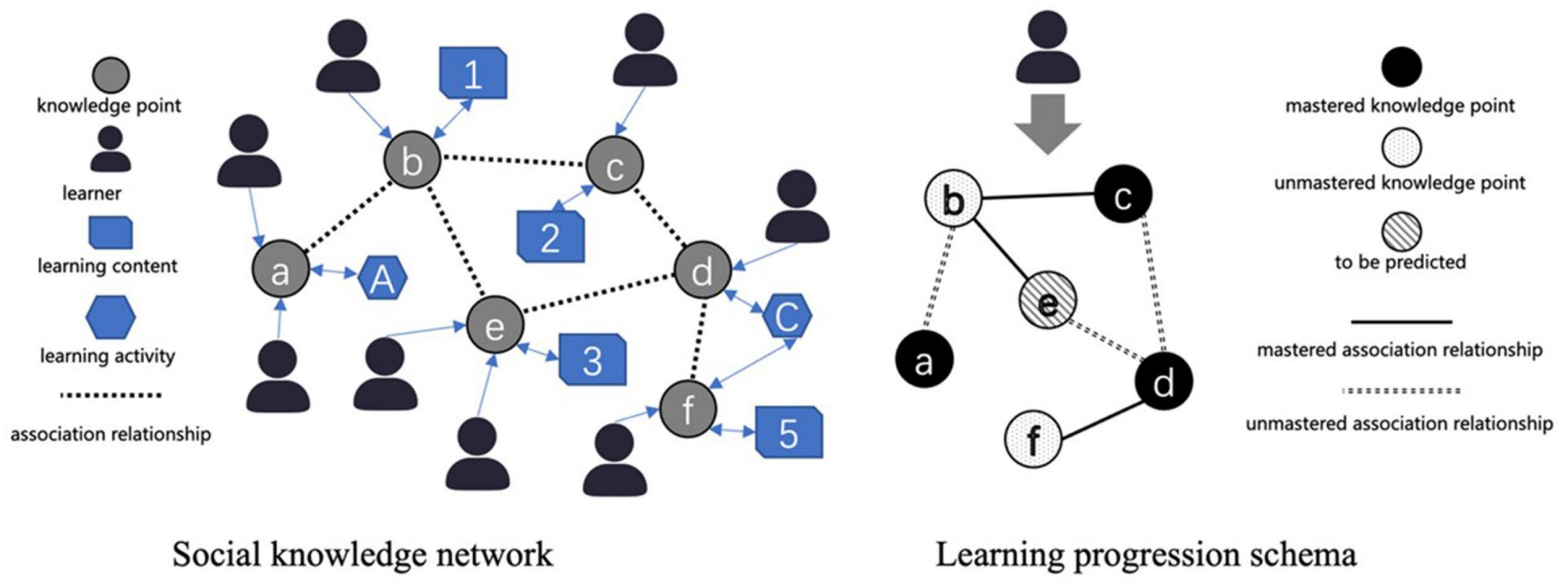
An illustration of social knowledge network and learning progression schema.

The dynamic knowledge network visualization component creates an interactive representation of course knowledge structures through multiple relationship types. The system represents connections between curriculum knowledge points, associations between knowledge points and learning resources, and interactions between learners and knowledge components. The system begins with expert-annotated associations between curriculum topics and knowledge points based on teaching outlines. As learners interact with the course, the system incorporates their interaction data to generate a dynamic social knowledge network. Specifically, this network uses the SK graph which is a collection of Subject Knowledge (SK) points and their parent–child relationships as its underlying structure. It dynamically renders a learner’s cognitive state (e.g., well known, unknown) for each node using different colors, and represents their understanding of the relationships between concepts using full or broken lines. This network reveals not only the conceptual relationships but also identifies influential learners associated with specific knowledge points, creating additional pathways for knowledge acquisition and peer learning. The visualization also includes a personal learning progression schema that displays each student’s knowledge structure development and mastery status. It is worth noting that learners do not directly see the progression of all other students. Instead, the dynamic recommendation module (Wan and Yu, 2020) calculates the similarity of the learning cognitive map between the current learner and others. It then recommends the most suitable learning peer based on this similarity, along with the learning resources associated with that peer. Figure 1 shows an illustration of social knowledge network and learning progression scheme. This individualized view helps learners understand their current state of knowledge and adjust their learning strategies accordingly. Our previous research has demonstrated the effectiveness of this approach in supporting self-regulated learning (Wan and Yu, 2020).

### 3.2 Adaptive activity pathway generating component

**Figure 2:**
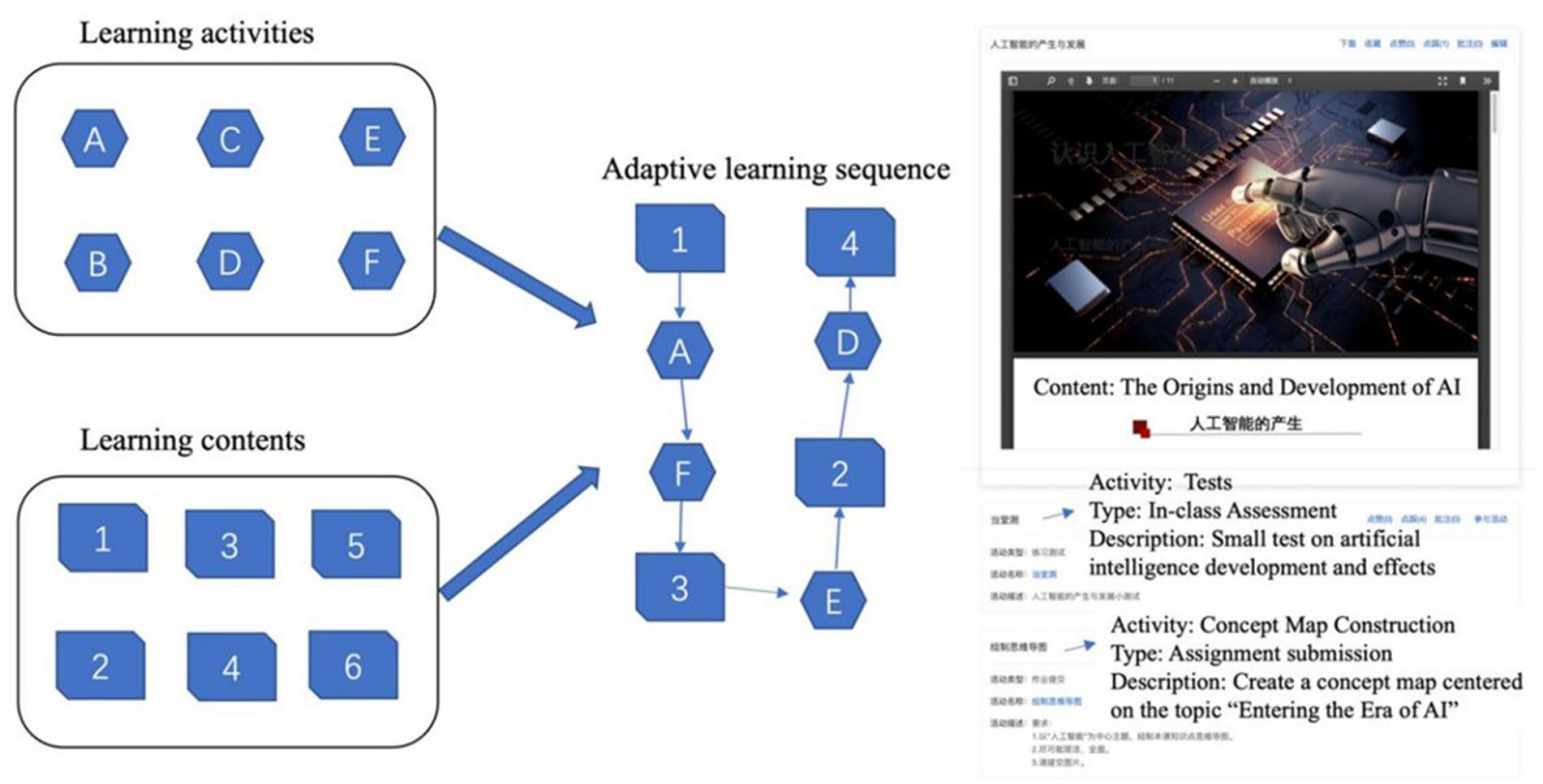
An illustration of adaptive learning pathways.

The second component addresses the challenge of fragmented learning experiences by creating explicit connections between learning content and activities. Unlike traditional approaches that present content and activities separately, our system integrates them through structured pathways. Course creators and collaborators can select from various activity types to achieve different learning objectives. These activities are then systematically linked to relevant learning content, forming coherent individual learning sequences. Specifically, the pathways are built upon a directed acyclic prerequisite diagram, which is automatically generated by applying the Apriori algorithm to association information and learners’ online learning scores, retaining rules with the highest associated degree (Wan and Yu, 2020). Moreover, adaptation occurs when a learner studies a specific SK. The system dynamically calculates the similarity of the learning cognitive map between that learner and others, based on the cognitive states of the SK’s brothers, parent, and children SKs. It then recommends the learning path, learning content, and learning activities associated with the most similar learning peer. This process is recalculated for each new SK, enabling continuous dynamic adaptation. See Figure 2 for an illustration. This integration promotes deeper interaction with content and higher-level cognitive engagement. The system continuously adapts these pathways based on several factors. It considers the relationships between learning activities and knowledge points, monitors student progress and performance, accounts for the natural progression of course concepts, and incorporates activity completion data from the learning community.

### 3.3 Integration of the components

The two components work synergistically to support learning. The knowledge network visualization helps learners understand the broader context of their learning, while the adaptive activity pathway component guides them through specific learning experiences. Together, they create a comprehensive support structure that addresses both knowledge organization and learning progression. When students access the course homepage, they can view the current unit’s knowledge network, which updates based on learning community interactions. The visualization helps them track their progress and identify areas needing attention. Simultaneously, the adaptive activity pathway component provides structured access to course content and resources, recommending relevant materials based on the student’s current learning focus and progress.

This integrated approach differs from traditional scaffolding in several ways. It provides dynamic rather than static support and creates explicit connections between knowledge organization and learning activities. The approach supports the development of higher-order thinking through structured progression while incorporating social learning aspects through the knowledge network. Its core function is to represent a learner’s knowledge structure and cognitive state visually and to provide dynamic recommendations for learning resources (i.e., learning content, learning activity, learning path, and learning peer).

## 4 Research methodology

### 4.1 Participants

The study involved 60 students from two parallel classes in Grade 1 at Y Middle School in northern China. Students ranged in age from 15 to 17 years. The experimental group consisted of 30 students (12 girls and 18 boys), while the control group included 30 students (15 girls and 15 boys). All participants possessed prior online learning experience and demonstrated proficiency in computer-based tasks such as concept mapping and online questionnaire completion. We got the informed consent from the students when we did the research in the pre-test questionnaire. All the students agreed to participate in the study.

### 4.2 Experimental procedure

**Figure 3:**
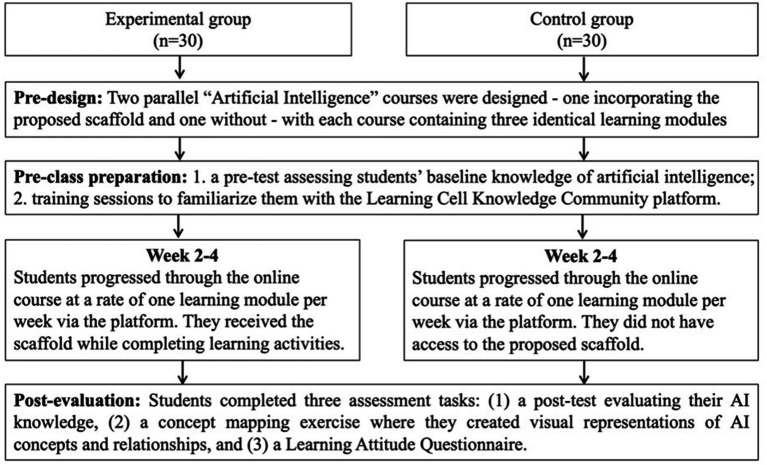
Overview of the experimental procedure and assessment timeline.

The experiment spanned 4 weeks, following a structured implementation process (see Figure 3). During the first week, all students completed a pre-test assessing their knowledge of artificial intelligence. Following the pre-test, all participants received training on the basic functions of the Learning Cell Knowledge Community (LCKC) platform from their course teacher in the computer classroom, then students practice using the platform on their computers. Moreover, students in the experimental group received additional training on the integrated scaffolding approach, specifically focusing on the knowledge network visualization and activity pathway components.

Over the subsequent 3 weeks, both groups studied three topics within the “Entering the Era of Artificial Intelligence (AI)” curriculum: understanding AI, experiencing AI, and the application and impact of AI. Each topic required one 40-min class session. The curriculum covers 18 core knowledge points, including the definition, development and characteristics of artificial intelligence, the principles of artificial intelligence technologies such as voice interaction and image recognition, and the application fields of artificial intelligence. Throughout this period, all students completed three online practice tests, one commentary activity, and three subjective questions about AI.

The experimental group accessed the course through our integrated scaffolding approach. They could view and interact with the knowledge network visualization on the course homepage, with the structured knowledge network updating based on student needs and teaching requirements. These students also received integrated content and activity resources, with structured activities organized around specific knowledge points and relevant multi-modal resources (including graphics, hyperlinks, and videos) provided based on activity themes. In contrast, the control group accessed the same course content through the standard LCKC interface without the scaffolding. They did not have access to the knowledge network visualization or the structured activity pathways, nor did they receive the integrated resource recommendations.

At the conclusion of the study, all students created concept maps focused on “Artificial Intelligence.” The task required them to include basic elements such as concepts, connections, hierarchy, and propositions. Students also provided concept definitions and examples, with the option to add supplementary terms as needed. All participants had received prior instruction in concept mapping techniques. Finally, students completed a post-test on AI knowledge and filled out a questionnaire about their attitudes toward AI.

### 4.3 Assessment instruments

#### 4.3.1 AI knowledge test

The AI knowledge test, developed in collaboration with the information technology director and subject experts, consisted of nine multiple-choice questions. Each question was worth 10 points, totaling 90 points. The first three questions examined AI-related concepts, while the remaining six focused on implementation technologies and application domains. Both groups completed identical tests before and after the experiment, with correct answers and explanations provided only in the post-test.

#### 4.3.2 Concept map assessment scale

The concept map served as a visual knowledge organization tool to represent complex knowledge structures and hierarchical relationships between various concepts (Davies, 2011). This visualization method helps deepen learners’ understanding and promotes deep learning (Dmoshinskaia et al., 2021). We developed our assessment scale based on Novak et al.’s (1984) scoring system and Ma et al.’s (2021) scoring framework. The scale evaluated four dimensions: concept quality, concept connections, hierarchical structure, and concept examples. Table 1 shows detailed concept map assessment rubrics.

**Table 1:** Concept map assessment scale.

Component	Description	Scoring criteria
Concept	Each concept represents a distinct AI-related term that is clearly defined and directly relevant to the topic. Concepts must be expressed as specific terms or phrases rather than general statements.	One point for each valid and relevant concept
Connection	Directional arrows connect pairs of concepts, with linking words or phrases that accurately describe the relationship between the concepts. The relationship must be valid and meaningful within the context of AI.	One point for each valid and accurately labeled connection
Hierarchy	The concept map demonstrates clear hierarchical organization, with broader, more general concepts at higher levels and increasingly specific concepts at lower levels. Each level shows appropriate subordination of ideas.	Five points for each valid hierarchical level that demonstrates proper concept progression
Examples	Specific real-world instances, applications, or cases are provided to illustrate concepts. Examples must be concrete, accurate, and demonstrate understanding of the concept being illustrated.	One point for each relevant and accurate example

For reliability assessment, two researchers independently graded 20 randomly selected concept maps from both groups. Analysis using IBM SPSS Statistics 21 yielded a Kappa coefficient of 0.83, indicating strong inter-rater reliability (Hwang and Chang, 2020). Subsequently, one researcher completed the grading of the remaining concept maps.

#### 4.3.3 Learning attitude questionnaire

Based on Defleur and Westie’s (1958) theory that deep learning generates useful knowledge and increased topic interest, we modified their scientific infatuation questionnaire to assess students’ engagement with AI topics. The questionnaire examined attitudes through explicit behavior (such as AI activity participation), cognitive evaluation (such as perceived importance of AI knowledge), and emotional response (such as curiosity about AI principles). The instrument contained 21 five-point Likert-scale items, with responses ranging from “strongly disagree” (1) to “strongly agree” (5). Each dimension (behavior, cognition, and emotion) included seven questions. The overall Cronbach’s *α* reached 0.94, with individual dimension reliability coefficients exceeding 0.8 for both experimental and control groups, indicating strong internal consistency (see Table 2).

**Table 2:** Internal consistency reliability coefficients by dimension and group.

Dimension	Experimental group (*n* = 30)	Control group (*n* = 30)
Behavior	0.86	0.92
Cognition	0.94	0.95
Emotion	0.94	0.95

#### 4.3.4 Student response analysis

To evaluate thinking levels in different scaffolding contexts, we analyzed students’ responses to three open-ended questions: “Q1: What do you understand about AI?,” “Q2: Will you feel pressure if your classmate is an AI in the future?,” and “Q3: Could machines replace humans in emotional expression through poetry?” The analysis employed the Structure of the Observed Learning Outcome (SOLO) classification framework (Aronshtam et al., 2021).

We developed a coding schema based on the SOLO taxonomy, which consists of five levels of increasing structural complexity: Pre-structural (responses lacking coherent understanding), Uni-structural (responses focused on a single relevant aspect), Multi-structural (responses incorporating multiple but unconnected elements), Relational (responses demonstrating integrated understanding with meaningful connections), and Extended Abstract (responses showing comprehensive understanding with novel applications and broader implications). Table 3 shows the coding schema in detail. To ensure the scientific nature of the research, we anonymized all the data. The two trained researchers first examined and conducted an in-depth analysis of the coding scheme, indicators, and samples. They randomly selected the responses of 10 students as samples and discussed the coding process. Then, two researchers independently coded all student responses to the three questions. The coding achieved a Cohen’s kappa value of 0.81, indicating high reliability (Van Hoe et al., 2024). The researchers resolved any coding discrepancies through discussion to reach consensus.

**Table 3:** Coding schema for student response analysis using SOLO taxonomy.

SOLO Level	Description
Pre-structural	Student response lacks coherent understanding of the task, containing irrelevant information or unconnected concepts. No meaningful engagement with the AI-related question is demonstrated.
Uni-structural	Student response focuses on a single relevant aspect of AI, demonstrating basic understanding but missing other important elements. The answer relies on one piece of information without broader context.
Multi-structural	Student response includes multiple relevant aspects of AI but presents them as separate, unconnected pieces of information. Relationships between different elements are not established, and integration is lacking.
Relational	Student response integrates multiple aspects of AI knowledge into a coherent framework, establishing meaningful connections between concepts. The answer demonstrates understanding of how different elements relate to and influence each other
Extended abstract	Student response demonstrates comprehensive understanding by abstracting principles from integrated knowledge, generating novel hypotheses, and applying concepts to new contexts. The answer extends beyond given information to create broader implications or applications of AI concepts.

Adapted from Aronshtam et al. (2021).

## 5 Results and discussion

The study findings revealed significant effects of the activity-enhanced scaffolding for knowledge structure representing across three key dimensions: learning performance, learning attitudes, and thinking levels. This section presents and discusses these results in relation to our research questions.

### 5.1 Impact on learning performance

The analysis of learning performance encompassed both knowledge acquisition through standardized testing and knowledge organization through concept mapping. These complementary measures provided comprehensive insights into the effectiveness of the scaffolding approach.

#### 5.1.1 Knowledge acquisition assessment

Table 4 presents the comparison of AI knowledge test scores between the experimental and control groups at both pre-test and post-test. Pre-test analysis revealed no significant differences between the experimental and control groups (*t* = 0.67, *p* = 0.503), the effect size Cohen’s *d* value is 0.168 < 0.2, indicating comparable baseline knowledge. After the experiment, post-test results showed significantly higher scores in the experimental group compared to the control group (*t* = 2.22, *p* = 0.030), and the Cohen’s d value is 0.569 > 0.5, indicating a moderate effect strength.

**Table 4:** Comparison of AI knowledge test scores between groups at pre-test and post-test.

Iteam	Group	*N*	*M*	*SD*	*t*
Pre-test	Experiment group	30	54.55	15.83	0.67
	Control group	30	51.94	15.15	
Post-test	Experiment group	30	84.33	6.79	2.22*
	Control group	30	78.44	12.98	

*p < 0.05. M, Mean; SD, Standard deviation.

Preliminary analyses confirmed that the data met the assumptions for ANCOVA (Analysis of Covariance). The Kolmogorov–Smirnov test indicated normal distribution across all datasets (*p* > 0.05), and the homogeneity of regression assumption was satisfied (*F* = 0.185, *p* = 0.668). Table 5 presents the results of the one-way ANCOVA. ANCOVA analysis, controlling for pre-test performance, confirmed the significant effect of the scaffolding approach (*F* = 4.97, *p* = 0.030, η^2^ = 0.078). The experimental group demonstrated higher adjusted mean scores (*M* = 84.38) compared to the control group (*M* = 78.40), with a medium to large effect size suggesting practical significance (De Ruyck et al., 2023).

**Table 5:** ANCOVA results for ai knowledge post-test scores.

Group	Mean	*SD*	Adjusted Mean	*SE*	*F*	*η* ^2^
Experiment group	84.33	6.79	84.38	1.93	4.97*	0.078
Control group	78.44	12.98	78.40	1.86		

*p < 0.05.

The superior performance of the experimental group can be attributed to the scaffold’s dual support mechanisms. The visualized social knowledge network enabled students to comprehend the course’s overall knowledge structure and monitor their learning progress, facilitating timely adjustments to their learning strategies. This finding aligns with Wang et al. (2019), who demonstrated that visual group awareness tools enhanced learning performance by making individual progress and peer interactions visible to learners. Furthermore, the scaffold’s integration of knowledge points with learning content and activities helped students identify and access relevant resources based on their mastery level. This supports more effective knowledge acquisition and retention.

#### 5.1.2 Knowledge organization capability

Concept map analysis revealed significantly higher scores in the experimental group (*M* = 42.00, *SD* = 2.46) compared to the control group (*M* = 38.31, *SD* = 3.38; *t* = 5.22, *p* < 0.001), and the effect size d value is 1.248 > 0.8, indicating a large effect strength. The difference suggests that the activity-enhanced scaffolding enhanced students’ ability to organize and connect knowledge meaningfully, aligning with previous findings on the benefits of knowledge visualization for conceptual understanding (Liu et al., 2020). These results support findings by Wang et al. (2019) on the effectiveness of visual knowledge representation. The dynamic knowledge network visualization enabled students to comprehend the broader knowledge structure. Moreover, the integrated activity pathways facilitated systematic knowledge construction by connecting learning content with relevant activities (Wu et al., 2023).

### 5.2 Effects on learning attitudes

Analysis of learning attitudes revealed significant positive effects of the scaffolding across all three measured dimensions: behavioral engagement (*t* = 2.26, *p* = 0.027), cognitive evaluation (*t* = 2.39, *p* = 0.020), and emotional response (*t* = 2.90, *p* = 0.005) (see Table 6), and the effect size d value of these three measured dimensions are 0.877, 5.761, and 14 respectively, all indicating a relatively strong effect. The experimental group demonstrated consistently higher mean scores across all dimensions compared to the control group. For behavioral engagement, the experimental group scored higher (*M* = 4.21) than the control group (*M* = 3.71). Similarly, in cognitive evaluation, the experimental group showed stronger results (*M* = 4.60) compared to the control group (*M* = 4.07). The pattern continued in emotional response, where the experimental group again achieved higher scores (*M* = 4.50) than the control group (*M* = 3.73).

**Table 6:** Group differences in learning attitude dimensions.

Dimension	Group	*N*	*M*	*SD*	*t*	*p*
Behavior	Experiment group	30	4.21	0.74	2.26	0.027*
	Control group	30	3.71	0.32		
Cognition	Experiment group	30	4.60	0.12	2.39	0.020*
	Control group	30	4.07	0.05		
Emotion	Experiment group	30	4.50	0.06	2.90	0.005**
	Control group	30	3.73	0.05		

These results suggest that the scaffolding approach enhanced students’ overall engagement with the learning material. The dynamic knowledge visualization component likely reduced learning disorientation, while the structured activity pathways provided clear guidance for engagement, supporting previous findings on the relationship between scaffolding and learning motivation (Chang et al., 2018; Wu et al., 2023).

### 5.3 Impact on thinking levels

**Figure 4:**
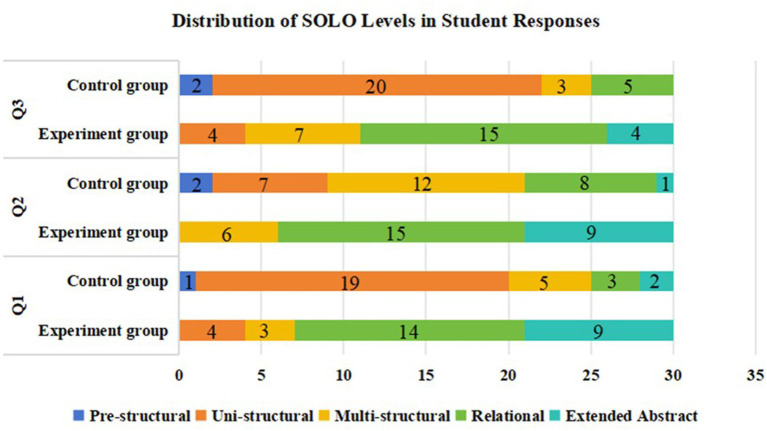
Distribution of SOLO levels in student responses.

Analysis of student responses using the SOLO taxonomy revealed substantial differences in thinking levels between groups on each question (see Figure 4). The experimental group exhibited higher levels of cognitive development, with 73.33% (66 responses) of responses reaching the relational or extended abstract levels, compared to only 21.11% (19 responses) in the control group. The experimental group’s responses were concentrated in higher-order thinking categories: 48.89% (44 responses) demonstrated relational thinking and 24.44% (22 responses) achieved extended abstract understanding. Lower percentages were observed for multi-structural thinking at 17.78% (16 responses) and uni-structural responses at 8.89% (8 responses). In contrast, the control group’s responses clustered in lower-order thinking categories, with uni-structural responses dominating at 51.11% (46 responses), followed by multi-structural responses at 22.22% (20 responses). Higher-order thinking was less prevalent in the control group, with relational responses at 17.78% (16 responses) and extended abstract responses at only 3.33% (3 responses). Furthermore, 5.56% (5 responses) of the control group’s responses remained at the pre-structural level, indicating minimal conceptual understanding.

The higher proportion of advanced thinking levels in the experimental group can be attributed to several key features of the structuring scaffold. First, the visualization of social knowledge networks helped students construct meaningful connections between different AI concepts and their applications. By providing a clear view of how knowledge components interrelate, the scaffold supported students in moving beyond isolated facts (uni-structural thinking) toward more integrated understanding (relational thinking). This aligns with previous findings by Chen and Tsao (2020), who demonstrated that visual representation of concept relationships facilitates deeper cognitive processing.

The scaffold’s integration of learning content with associated activities likely contributed to the experimental group’s superior performance in relational (48.89%) and extended abstract (24.44%) thinking. When students could see explicit connections between concepts and their practical applications through structured activities, they were better equipped to form sophisticated mental models of AI topics. This structured approach to knowledge organization has been shown to upgrade curriculum coherence and promote higher-order thinking development (Yu et al., 2017; Wu et al., 2023).

The control group’s concentration in uni-structural responses (51.11%) suggests that without scaffolding support, students tended to process information in a fragmentary manner, focusing on isolated facts rather than integrated understanding. This pattern exemplifies the common challenge in online learning where students struggle to connect discrete pieces of knowledge into coherent frameworks when structured guidance is absent. The relatively low percentage of extended abstract responses in the control group (3.33%) further indicates that traditional online learning approaches may be insufficient for fostering the highest levels of cognitive development.

## 6 Conclusion

This study addressed the challenges of online learning by developing and evaluating an activity-enhanced scaffolding for knowledge structure representing that combines dynamic knowledge network visualization with adaptive activity pathways. Through a quasi-experimental study involving 60 middle school students, we found that this approach significantly enhanced learning outcomes across multiple dimensions. Students using the scaffolding approach demonstrated superior performance in knowledge acquisition and organization, showed more positive learning attitudes, and achieved higher levels of cognitive engagement. The findings contribute to both theoretical understanding and practical applications in online learning. Theoretically, the study extends existing scaffolding theories by demonstrating the effectiveness of integrating knowledge visualization with structured learning pathways. Practically, the results provide concrete guidelines for designing online learning environments that better support student progression and deeper learning, particularly in information technology education at the secondary level. Furthermore, the superior performance of the experimental group also reflects to a certain extent that this scaffold method is conducive to transforming cutting-edge scientific and technological knowledge into an important carrier for promoting students’ cognitive development, providing the possibility for cultivating students’ digital literacy necessary to adapt to the intelligent era.

Several limitations should be considered when interpreting these results. First, a small sample size may reduce the statistical testing power and limit the generalizability of the conclusion. Meanwhile, the study’s relatively short duration of 4 weeks may not fully capture the long-term effects of the scaffolding approach on learning outcomes. Second, the system begins with expert-annotated associations between curriculum topics and knowledge points based on teaching outlines. This process was time-intensive and could potentially limit scalability. Future research will consider introducing large models to reduce the time cost in this stage, which also provides certain inspirations for the integration of generative artificial intelligence into the teaching practices of primary and secondary schools. Third, while the approach demonstrated effectiveness in information technology education, its applicability across different subject areas remains to be verified. Additionally, the study’s focus on middle school students may limit the generalizability of findings to other educational levels. For future, the scaffolding is conducted in AI curriculum in middle school and how to use it in other system remains to be seen.

## Data Availability

The raw data supporting the conclusions of this article will be made available by the authors, without undue reservation.

## Footnotes

1
http://www.etc.edu.cn/
